# Pembrolizumab combined with low-dose cyclophosphamide and intra-tumoral injection of the toll-like receptor 4 agonist G100 in patients with advanced pretreated soft tissue sarcoma: results from the PEMBROSARC basket study

**DOI:** 10.1186/s13045-022-01377-2

**Published:** 2022-10-27

**Authors:** Mariella Spalato-Ceruso, Fanny Bouteiller, Jean-Philippe Guegan, Maud Toulmonde, Alban Bessede, Michèle Kind, Sophie Cousin, Xavier Buy, Jean Palussiere, François Le Loarer, Berengere Dadone-Montaudie, Marina Pulido, Antoine Italiano

**Affiliations:** 1 Early Phase Trials Unit, Department of Medicine, Institut Bergonié, 33000 Bordeaux, France; 2 Clinical and Epidemiology Department, Clinical Investigation Centre, Bordeaux, France; 3 Explicyte, Bordeaux, France; 4 Department of Imaging, Institut Bergonié, Bordeaux, France; 5 Department of Pathology, Institut Bergonié, Bordeaux, France; 6 Faculty of Medicine, University of Bordeaux, Bordeaux, France

## Abstract

Soft tissue sarcomas (STS) are heterogeneous mesenchymal tumors with limited therapeutic options in the advanced setting. Immune checkpoint inhibitors have been shown to have significant clinical activity in inflamed STS which are characterized by the presence of tertiary lymphoid structures (TLS). New strategies are needed to sensitize TLS-negative STS to immunotherapy. Engagement of the toll-Like Receptor 4 (TLR4) signal pathway contributes to the development of a favorable tumor microenvironment in solid tumors. G100 is a highly potent toll-like receptor 4 (TLR4) agonist. We hypothesized that intra-tumoral G100 would induce a robust local and potentially systemic anti-tumor immune response in the microenvironment of TLS-negative sarcoma, leading to improved response to PD1 inhibition. Twenty metastatic STS patients who had a superficial injectable lesion were treated with 50 mg of cyclophosphamide (CP) orally twice daily (1 week on and 1 week off), 200 mg of pembrolizumab intravenously on day 8 of a planned 21-day cycle and G100 20 µg one weekly intra-tumoral injection for at least 6 weeks and for a maximum of 12 weeks (1st injection one week before CP administration, ie. Day -7). Biopsies and blood were collected pre and post treatment. Of the 17 patients assessable for efficacy analysis, 2 were progression-free at 6 months, and the 6-month non-progression rate was 11.8% (95% CI: 1.5–36.4), indicating that the first endpoint of the study was not reached. In 8 patients, there was an increase in T-cell infiltration into tumor after treatment. The ratio CD8/Fox-P3 + CD4 on treatment decreased in 11 cases out of 14 suggesting a predominant induction of Treg. Soluble PDL1 levels at baseline were also with adverse outcome. G100 appears to modulate the tumor microenvironment with significant infiltration of T cells. However, clinical activity in combination with PD1 inhibition was limited and no clear correlation was observed between tumor shrinkage and increased inflammation. TLR4 stimulation might have both antitumor and pro-tumor consequences.

*Trial registration*: This study was registered with ClinicalTrial.gov, number NCT02406781.

**Supplementary Information:**

The online version contains supplementary material available at 10.1186/s13045-022-01377-2.

To the editor

We report here the results of the cohort of the PEMBROSARC study [1] aiming to assess the safety, efficacy, and immunologic effects of the TLR4 agonist G100 administered intra-tumorally (IT) combined with pembrolizumab in patients with advanced soft-tissue sarcomas (STS).

Patients received 50 mg of cyclophosphamide (CP) orally twice daily (1 week on and 1 week off), 200 mg of pembrolizumab intravenously on day 8 of a planned 21-day cycle and G100 20 µg one weekly intra-tumoral injection for at least 6 weeks and for a maximum of 12 weeks (1st injection one week before CP administration, i.e., Day -7). Eligibility criteria and methods are detailed in Additional file 1: Methods.

Between February 8, 2019 and December 3, 2020, 20 patients were included in the study (Additional file 1: Figure S1). Seventeen patients were assessable for the primary efficacy endpoint, which was the 6-month non-progression rate (Additional file 1: Figure S1) Baseline patient characteristics are listed in Additional file 1: Table S1.

After a median follow-up of 15.6 months [95% confidence interval (CI): 13.6–17.9], one patient was (13.3%) still receiving treatment. Discontinuation was related to disease progression in 18 cases (73%) and investigator decision for one patient (15.5%) (Additional file 1: Figure S1).

Two patients were progression-free at 6 months, and the 6-month non-progression rate was 11.8% (95% CI: 1.5–36.4), indicating that the first endpoint of the study was not reached. Of the 17 patients eligible and assessable for efficacy, 5 (29.4%) had tumor shrinkage resulting in partial response in 1 case (5.9%) and stable disease in 4 cases (23.5%) (Fig. 1A). The best response was partial response for one patient [1 undifferentiated pleomorphic sarcoma], stable disease for 4 patients [1 epithelioid sarcoma, 1 leiomyosarcoma, 2 dedifferentiated liposarcomas] and progressive disease for 12 patients [1 angiosarcoma, 2 solitary fibrous tumor, 8 leiomyosarcoma, 1 pleomorphic rhabdomyosarcoma].

**Fig. 1:**
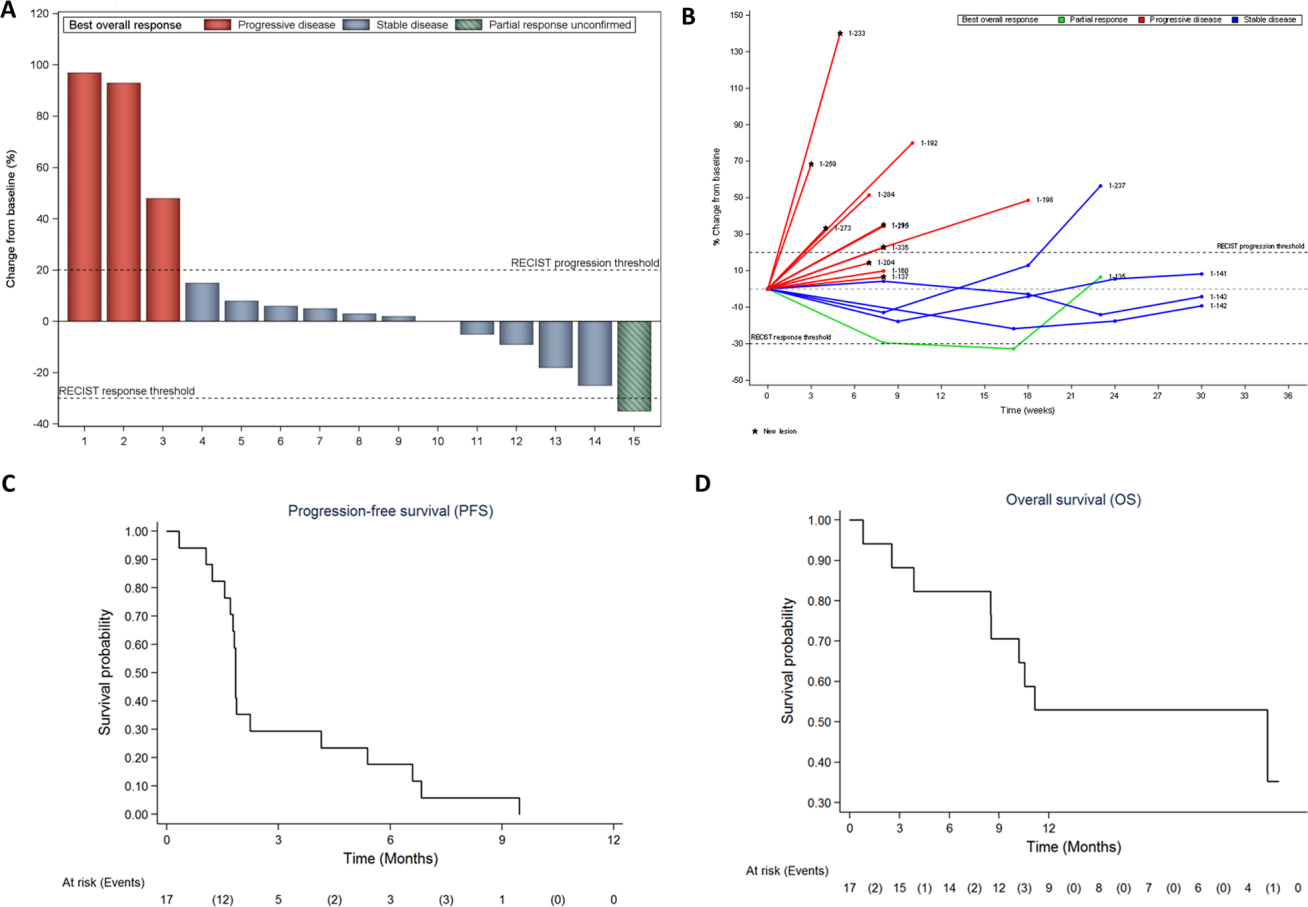
Waterfall plots of tumor response **A** spider plots **B** and Kaplan–Meier curves of progression-free **C** and overall survival **D** Only patients with available tumor assessments after central review at data cutoff are shown. Changes in tumor size were centrally assessed by blinded independent review according to Response Evaluation Criteria in Solid Tumors (RECIST) 1.1. Maximum change in sum of diameters from baseline is shown on the waterfall plots (**A**); time on treatment is shown on spider plots (**B**)

The median follow-up time was 11.2 months (95% CI: 7.6–11.7 months). Median PFS was 1.8 months (95% CI: 1.5–5.0 months) (Fig. 1C). The 6-month and 1-year PFS rates were 11.8% (95% CI: 2–31.2%) and 0%, respectively. Additionally, the median overall survival (OS) was 10.6 months (95% CI: 8.5 months – NA) (Fig. 1D). After treatment discontinuation, 18 patients received additional lines of systemic therapies (median: *n* = 1, range 1–4). The most frequent regimens were gemcitabine combined with dacarbazine (*n* = 7) and gemcitabine (*n* = 4). The safety analysis was reported in Additional file 1: Table S2.

We have recently demonstrated that a subset of STS is characterized by the presence of tertiary lymphoid structures (TLS) and that TLS status represent a reliable biomarker to select patients with advanced STS for treatment with immune checkpoint inhibitors [2, 3]. All the patients enrolled in this study had negative TLS-status and can be considered as “cold” STS [2]. We have evaluated the ability of G100 to alter the tumor microenvironment of STS in sequential tumor samples obtained at baseline and at cycle 2 Day 1 treatment in 14 patients. Multiplex immunofluorescence (IF) analysis demonstrated increase in intra-tumoral CD8 + T and CD4 T-cells infiltration post-G100 therapy in 7 (50%) and 8 patients (57%), respectively (Additional file 1: Figure S2). While these data suggest that G100 was able to increase intra-tumoral inflammation in a subset of patients, there was no clear correlation between baseline or dynamic expression status and responses in this cohort. Since Treg represent an important subset of CD4 + T cells and a major obstacle for the elimination of tumors by immune cells, we investigated the ratio of CD8 + /Fox-P3 + CD4 T cells. Strikingly, the ratio CD8/Fox-P3 + CD4 on treatment decrease in 11 cases out of 14 suggesting a predominant induction of Treg.

We also found that high circulating soluble programmed death-1 ligand (sPD-L1) was the sole protein significantly associated with worse PFS and OS (Additional file 1: Tables S3 and S4).

This study represents the first investigation of the TLR4 agonist G100 administered IT in combination with intravenous PD1 antagonist in patients with advanced cold STS. Analysis of the sequential biopsies revealed that IT G100 resulted in CD4 and CD8 T cell increase in more than 50% of patients and particularly for the CD4. These results are in line with those of previous study which investigated the effect of intratumorally injection of G100 in 15 metastatic STS patients with superficial lesions and which showed that G100 pushed the tumor microenvironment into a more inflammatory state, driven largely by an increase in T cell infiltration [4]. However, as observed in our study this increase in lymphocytic infiltration did not translate into substance clinical benefit. Moreover, no clear correlation was observed between tumor shrinkage and increased inflammation. One potential explanation for this observation may be related to the fact that TLR4 stimulation might have both antitumor and pro-tumor consequences. For instance, it has been shown that TLR4 activation can result in enhanced regulatory T-cell proliferation and suppressor function favoring tumor development [5]. Although we observed an increased density of CD8 + and FoxP3 + CD4T post-treatment in a significant proportion of the patients included in our study, the CD8/Foxp3 CD4 T cell decreased in all patients but 3 suggesting a predominant induction of Treg by G100 that may explain the limited clinical activity.

Overall, our results demonstrate that TLR4 agonist may have significant impact of tumor microenvironment of TLS-negative STS. However, our findings showing an impact of G100 on Treg-cell tumor infiltration indicate that further studies are needed to clarify the role of TLR4 agonist on tumor microenvironment.

## Supplementary Information

**Additional file 1**: Supplementary Methods, Tables and Figures.

## Data Availability

The datasets generated during and/or analyzed during the current study are not publicly available due to the clinical and confidential nature of the material but can be made available from the corresponding author on reasonable request.

## Abbreviations

CP: Cyclophosphamide
PFS: Progression-free survival
Soluble programmed death-1 ligand: Soluble programmed death-1 ligand
STS: Soft-tissue sarcoma
TK: Thymidine kinase
OS: Overall survival
