# Supplementary material for: Pembrolizumab combined with low-dose cyclophosphamide and intra-tumoral injection of the toll-like receptor 4 agonist G100 in patients with advanced pretreated soft tissue sarcoma: results from the PEMBROSARC basket study
Source: J Hematol Oncol. 2022 Oct 27;15:157. doi: 10.1186/s13045-022-01377-2 (PMC9609223; doi:10.1186/s13045-022-01377-2)
Supplement: Supplementary file 1 — Additional file 1: Supplementary Methods, Tables and Figures. [file 13045_2022_1377_MOESM1_ESM.doc]

# PATIENTS AND METHODS

***Trial design and oversight***

PEMBROSARC is a multi-cohort phase 2 study enrolling patients with advanced sarcomas in 7 centers from the French Sarcoma Group. The study was based on Simon’s optimal two-stage design and conducted in accordance with the Declaration of Helsinki and Good Clinical Practices. All patients provided written informed consent. Confirmation of disease progression based on central review of two imaging obtained at less than a 6-months interval was mandatory at inclusion. Patients received 50 mg of cyclophosphamide (CP) orally twice daily (1 week on and 1 week off), 200 mg of pembrolizumab intravenously on day 8 of a planned 21-day cycle and G100 20 µg one weekly intra-tumoral injection for at least 6 weeks and for a maximum of 12 weeks (1st injection one week before CP administration, ie. Day -7). As required by the French regulation, the protocol was centrally approved by a central IRB (the Comité de Protection des Personnes Sud-Ouest et Outre Mer III,Bordeaux, France) which reviewed the appropriateness of the clinical trial protocol as well as the risks and benefits to study participants. All patients provided written informed consent. Safety and efficacy data were reviewed by an independent data monitoring committee.

***Patients***

Main eligibility criteria included: Age ≥ 18 years; Eastern Cooperative Oncology Group (ECOG) performance status ≤ 1 ;Histological confirmation of soft-tissue sarcoma by central review ; Documented progression according to RECIST criteria. Progression on the last line of treatment should be confirmed by central review with two radiological assessments identical (CT scans or MRI) obtained at less than 6 months interval within the 12 months before inclusion, except for patients with metastatic disease diagnosed less than 6 months before inclusion; locally advanced or metastatic disease with at least one injectable lesion; adequate hematological, renal, metabolic, and hepatic functions. Full list of eligibility criteria is available in the protocol

***Study treatment***

Patients received 50 mg of cyclophosphamide (CP) orally twice daily (1 week on and 1 week off), 200 mg of pembrolizumab intravenously on day 8 of a planned 21-day cycle and G100 20 µg one weekly intra-tumoral injection for at least 6 weeks and for a maximum of 12 weeks (1^st^ injection one week before CP administration, ie. Day -7). Patients discontinued treatment if one of the following occurred: the patient made the decision to withdraw or there was unacceptable toxicity, disease progression according to RECIST 1.1 criteria, undercurrent illness, or changes in patient condition preventing further treatment at the discretion of the investigator.

***Response assessment and toxicity***

Tumor assessment was performed at week 9, week 18 , week 24 and every 6 weeks thereafter. Response was determined according to RECIST 1.1 guidelines after blinded central imaging review. Toxicities were assessed continuously according to Common Terminology Criteria for Adverse Events v.4.0.

***TLS screening***

All cases were submitted to central histological review for confirmation of the diagnosis of STS. The TLS status was assessed, as previously described (Reference 17 in the main text). Staining of tumor samples and tonsils (France Bank Tissue, as positive controls) was performed with a Dako Autostainer (Agilent Technologies, Santa Clara, CA, USA). Chromogenic detection of TLSs was performed using HighDef Red IHC chromogen (AP; ADI-950-140-0030; Enzo Life Sciences, Farmingdale, NY, USA) for CD20 and Permanent HRP Green (ZUC070-100; Zytomed Systems, Berlin, Germany) for CD3. Nuclei were counterstained with hematoxylin (S3301; Dako; Agilent Technologies). After mounting with EcoMount (EM897L; Biocare Medical, Pacheco, CA, USA), the slides were scanned with a NanoZoomer (Hamamatsu Photonics, Hamamatsu, Japan). Halo 10 software was used to create a first layer to define the tumor area as identified by a pathologist on the hematoxylin and eosin slide. A classifier was used to detect TLSs with a surface area ≥60,000 µm² and create a second layer around these structures. CD3+ and CD20+ cells were counted inside TLSs and in the tumour area using an algorithm. Patients were enrolled in the trial when at least one CD3+/CD20+ aggregate containing >700 cells was detected in the tumor.

***Multiplex-IHF assay***

Multiplexed immunohistofluorescence was performed using the following panel CD8/GzmA/CD4/Foxp3/CD56 as previously described. The following antibodies were used: CD8 (C8/144B, Dako: Agilent Technologies), GzmA (EPR20161, Abcam, Cambridge, UK), CD4 (SP35, Ventana), Foxp3 (236A/E7, Abcam), CD56 (MRQ-42, Cell Marque).

***Plasma proteomics***

Proteome analysis has been performed thanks to the Olink Proximity Extension
Assay (PEA) (Olink Proteomics AB, Uppsala, Sweden). Briefly, plasma samples
were assessed using the Olink® Target 96 Inflammation panel (Olink
Proteomics AB, Uppsala, Sweden) according to the manufacturer's
instructions. The assay was quantified on the microfluidic real-time PCR
instrument Biomark HD (Fluidigm). Data were quality controlled and
normalized using an internal extension control and an inter-plate control,
to adjust for intra- and inter-run variation. The final assay read-out is
presented in Normalized Protein eXpression (NPX) values, which is an
arbitrary unit on a log2-scale where a high value corresponds to a higher
protein expression.

***Statistical analysis***

The primary endpoint was 6-month non-progression according to RECIST 1.1 criteria, and a Simon’s two-stage design was used. This was computed as the number of patients alive and progression-free at six months, divided by the number of patients included in the population of patients evaluable for efficacy. To be considered in the population of patients evaluable for efficacy, patients had to meet the eligibility criteria and receive at least one infusion of pembrolizumab, one dose of CP and one IT administration of G100. As such, patients of the population of patients evaluable for efficacy who died before 6 months were counted in the denominator, but not in the numerator. To distinguish a favorable true non-progression rate of 40% from a null rate of 15% (with 90% power and 5% type I error), 29 eligible and assessable patients were needed. Using 3-month or 6-month PFSs as the principal endpoints for phase 2 trials enrolling patients with STS is an international recommendation from the Sarcoma Tumor Group of the EORTC. Based on these recommendations, a 6-month PFS rate of > or = 30% is considered as a reference value to suggest drug activity in the 1st line setting; for second-line therapy, a 3-month PFS rate of > or = 40% would suggest a drug activity, and < or = 20% would suggest inactivity. Following inclusion of the first 13 assessable patients, accrual could continue for a total of 29 patients if at least three non-progressions were observed. At the end of recruitment, at least eight non-progressions were needed to conclude that the investigational treatment had a meaningful effect. Secondary endpoints included the best overall response according to RECIST 1.1^7^ criteria, 1-year PFS, 1-year OS, safety, and correlations with the immunological characteristics of the tumors. PFS was defined as the time from the start of treatment to the time of progression or death (from any cause). OS was defined as the time from the start of treatment to death (from any cause) or the last patient contact. Patients who were alive and progression-free were censored at the date of last follow-up. All enrolled patients who received at least one dose of one of the investigational drugs were eligible for safety analyses. To be assessed for the primary efficacy endpoint, a subject had to meet the eligibility criteria and receive at least one dose of CP and one infusion of pembrolizumab. Descriptive statistics were used to characterize patients at study entry and report toxicities.

Survival rates were estimated using the Kaplan–Meier method. Patients were classified as “high” or “low” for the different immune cell subsets based on an optimal cut-point value computed using the “survminer” R package (https://cran.r-project.org/web/packages/survminer/index.html). Differences between groups were evaluated using the Wilcoxon-Mann-Whitney test for continuous variables. All comparisons performed for the translational analyzes were exploratory and hypothesis generating.

***Data Availability***

The datasets that support the findings of this study are not publicly available due to information that could compromise research participant consent. According to French/European regulations, any re-use of the data must be approved by the appropriate ethics committee. Individual participant data that underlie the results reported in this article can be shared upon request to the corresponding author (AI). Proposals may be submitted up to 36 months following article publication.

**TABLES**

| **Supplementary Table 1. Patient characteristics (n=20)** | |
| --- | --- |
| **Variable** |  |
| **Gender, n (%)** |  |
| Male | 10 (50.0) |
| Female | 10 (50.0) |
| **Age** |  |
| Median, years (range) | 66 (37–78) |
| **ECOG PS, n (%)** |  |
| 0 | 7 (35.0) |
| 1 | 13 (65.0) |
| **Histological subtype (%)​** |  |
| Angiosarcoma | 1 (5.0) |
| Well differentiated/dedifferentiated liposarcomas | 3 (15.0)​ |
| Leiomyosarcomas | 8 (40.0) |
| Solitary fibrous tumor | 3 (15.0) |
| Undifferentiated pleiomorphic sarcoma | 1 (5.0)​ |
| Epithelioid sarcoma | 1 (5.0) |
| Other histotypes* | 3 (15.0) |
| **Grade (%)** |  |
| 1 | 1 (5.0) |
| 2 | 7 (35.0) |
| 3 | 4 (20.0) |
| Not gradable | 2 (10.0) |
| Unknown | 6 (30.0) |
| **Stage, n (%)** |  |
| Locally advanced |  |
| Metastatic |  |
| **Prior lines of chemotherapy in the advanced setting n (%)​** |  |
| 0 | 0 (0.0) |
| 1​ | 14 (70.0) |
| 2​ | 6 (30.0) |
| > 2​ | 0 (0.0) |
| **Prior lines of immunotherapy n (%)​** |  |
| 0 | 0 (0) |
| **TLS status** |  |
| Positive | 0 (0.0) |
| Negative | 20 (100.0) |
| **Sites of injection** |  |
| Skin/soft-tissue | 16 |
| Pleural | 3 |
| Lymph node | 1 |

* Malignant Peripheral Nerve Sheath Tumor, pleomorphic rhabdomyosarcoma, myxoid liposarcoma

ECOG PS, Eastern Cooperative Oncology Group performance status; UPS, undifferentiated pleomorphic sarcoma.

| **Supplementary Table 2: Treatment-related adverse events (N=20 patients)** | | | | | | | | | | | | |
| --- | --- | --- | --- | --- | --- | --- | --- | --- | --- | --- | --- | --- |
| **AE term** | | **Maximal intensity (N=19)** | | | | | | | | | | |
|  |  | **Grade 1** | | | | **Grade 2** | | | | **Grade 3** | | |
|  |  | **n** | | **%** | | **n** | | **%** | | **n** | | **%** |
| Anemia | 2 | | 10.5 | | 2 | | 10.5 | | . | | . | |
| Vertigo | 1 | | 5.3 | | . | | . | | . | | . | |
| Hypothyroidism | 1 | | 5.3 | | . | | . | | . | | . | |
| Constipation | 2 | | 10.5 | | . | | . | | . | | . | |
| Diarrhea | 2 | | 10.5 | | . | | . | | . | | . | |
| Gastrointestinal pain | 2 | | 10.5 | | . | | . | | . | | . | |
| Nausea | 6 | | 31.6 | | 1 | | 5.3 | | . | | . | |
| Vomiting | 1 | | 5.3 | | 1 | | 5.3 | | . | | . | |
| Fatigue | 7 | | 36.8 | | 3 | | 15.8 | | . | | . | |
| Fever | 1 | | 5.3 | | . | | . | | . | | . | |
| Flu like symptoms | 1 | | 5.3 | | . | | . | | . | | . | |
| Localized edema | 1 | | 5.3 | | 1 | | 5.3 | | . | | . | |
| Malaise | 1 | | 5.3 | | 1 | | 5.3 | | . | | . | |
| Pain | 1 | | 5.3 | | . | | . | | . | | . | |
| Alanine aminotransferase increased | 2 | | 10.5 | | . | | . | | . | | . | |
| Alkaline phosphatase increased | 1 | | 5.3 | | . | | . | | . | | . | |
| Aspartate aminotransferase increased | 2 | | 10.5 | | 2 | | 10.5 | | . | | . | |
| Lymphopenia | 1 | | 5.3 | | 4 | | 21.1 | | 2 | | 10.5 | |
| Thrombocytopenia | 1 | | 5.3 | | . | | . | | . | | . | |
| Arthralgia | 1 | | 5.3 | | 1 | | 5.3 | | . | | . | |
| Tumor pain | 3 | | 15.8 | | 1 | | 5.3 | | . | | . | |
| Paresthesia | 1 | | 5.3 | | . | | . | | . | | . | |
| Somnolence | 1 | | 5.3 | | . | | . | | . | | . | |
| Pruritus | 1 | | 5.3 | | . | | . | | . | | . | |
| Rash maculo-papular | 5 | | 26.3 | | 1 | | 5.3 | | . | | . | |

| **Supplementary Table 3**. Association of baseline circulating marker levels  with progression-free survival. | | | | | | |
| --- | --- | --- | --- | --- | --- | --- |
|  | Symbol | pval | Median PFS  High value | Median PFS Low value | | Delta |
| 1 | PD-L1 | 0,000596 | 1,659138 | 4,13963 | -2,48049 | |
| 2 | EN-RAGE | 0,001059 | 1,741273 | 5,38809 | -3,64682 | |
| 3 | IL-17C | 0,001509 | 1,856263 | 1,215606 | 0,640657 | |
| 4 | CASP-8 | 0,003662 | 1,659138 | 2,234086 | -0,57495 | |
| 5 | Flt3L | 0,005327 | 2,053388 | 1,544148 | 0,50924 | |
| 6 | NT-3 | 0,00602 | 1,856263 | 1,215606 | 0,640657 | |
| 7 | IL-1 alpha | 0,006442 | 2,053388 | 1,708419 | 0,344969 | |
| 8 | CD5 | 0,013451 | 2,234086 | 1,626283 | 0,607803 | |
| 9 | IL2 | 0,017821 | 6,143737 | 1,839836 | 4,303901 | |
| 10 | OPG | 0,024461 | 5,453799 | 1,823409 | 3,63039 | |
| 11 | SCF | 0,024957 | 2,234086 | 1,7577 | 0,476386 | |
| 12 | CD6 | 0,026147 | 1,823409 | 6,833676 | -5,01027 | |
| 13 | TNFSF14 | 0,029604 | 1,790554 | 4,13963 | -2,34908 | |
| 14 | CCL19 | 0,029973 | 2,234086 | 1,7577 | 0,476386 | |
| 15 | TNFB | 0,031043 | 3,186858 | 1,806982 | 1,379877 | |
| 16 | FGF-19 | 0,032229 | 1,774127 | 3,186858 | -1,41273 | |
| 17 | CST5 | 0,06488 | 2,036961 | 1,708419 | 0,328542 | |
| 18 | CXCL1 | 0,06605 | 1,806982 | 3,186858 | -1,37988 | |
| 19 | CXCL11 | 0,069269 | 1,7577 | 1,87269 | -0,11499 | |
| 20 | LAP TGF-beta-1 | 0,070029 | 1,806982 | 2,053388 | -0,24641 | |
| 21 | CCL25 | 0,072765 | 1,790554 | 1,87269 | -0,08214 | |
| 22 | CXCL5 | 0,074712 | 1,806982 | 3,186858 | -1,37988 | |
| 23 | CDCP1 | 0,075103 | 1,823409 | 5,453799 | -3,63039 | |
| 24 | IL5 | 0,094453 | 1,806982 | 2,053388 | -0,24641 | |
| 25 | CD244 | 0,094684 | 1,806982 | 3,186858 | -1,37988 | |
| 26 | 4E-BP1 | 0,100827 | 4,336756 | 1,839836 | 2,49692 | |
| 27 | ARTN | 0,103082 | 2,036961 | 1,806982 | 0,229979 | |
| 28 | IL-20RA | 0,105025 | 1,839836 | 1,741273 | 0,098563 | |
| 29 | CD8A | 0,106838 | 1,87269 | 1,790554 | 0,082136 | |
| 30 | CCL3 | 0,108975 | 1,774127 | 2,036961 | -0,26283 | |
| 31 | NRTN | 0,112445 | 1,839836 | 1,774127 | 0,065708 | |
| 32 | CCL4 | 0,119622 | 1,790554 | 1,87269 | -0,08214 | |
| 33 | MCP-4 | 0,120096 | 1,839836 | 1,839836 | 0 | |
| 34 | MMP-1 | 0,12117 | 1,379877 | 1,87269 | -0,49281 | |
| 35 | OSM | 0,126392 | 4,320329 | 1,839836 | 2,480493 | |
| 36 | FGF-23 | 0,129891 | 1,839836 | 4,76386 | -2,92402 | |
| 37 | LIF | 0,130847 | 2,053388 | 1,839836 | 0,213552 | |
| 38 | IL13 | 0,134423 | 2,234086 | 1,823409 | 0,410678 | |
| 39 | SLAMF1 | 0,139569 | 2,036961 | 1,806982 | 0,229979 | |
| 40 | FGF-5 | 0,1408 | 1,839836 | 1,839836 | 0 | |
| 41 | IL-18R1 | 0,143114 | 2,036961 | 1,839836 | 0,197125 | |
| 42 | CXCL9 | 0,143807 | 1,823409 | 4,13963 | -2,31622 | |
| 43 | TSLP | 0,148299 | 1,839836 | 1,774127 | 0,065708 | |
| 44 | IL-10RB | 0,148758 | 1,856263 | 1,806982 | 0,049281 | |
| 45 | IL-15RA | 0,150376 | 1,823409 | 2,234086 | -0,41068 | |
| 46 | CXCL10 | 0,157989 | 1,708419 | 1,856263 | -0,14784 | |
| 47 | MCP-1 | 0,159545 | 1,839836 | 1,823409 | 0,016427 | |
| 48 | IL-12B | 0,164317 | 1,856263 | 1,806982 | 0,049281 | |
| 49 | IL-20 | 0,167097 | 1,839836 | 1,774127 | 0,065708 | |
| 50 | IL-24 | 0,177297 | 4,188912 | 1,839836 | 2,349076 | |
| 51 | CCL20 | 0,177436 | 1,839836 | 4,76386 | -2,92402 | |
| 52 | VEGFA | 0,178121 | 2,234086 | 1,839836 | 0,394251 | |
| 53 | TWEAK | 0,184145 | 1,823409 | 2,234086 | -0,41068 | |
| 54 | IL18 | 0,193685 | 1,839836 | 3,811088 | -1,97125 | |
| 55 | DNER | 0,197925 | 1,839836 | 4,024641 | -2,1848 | |
| 56 | IL-2RB | 0,199289 | 2,036961 | 1,839836 | 0,197125 | |
| 57 | MCP-2 | 0,199354 | 3,613963 | 1,839836 | 1,774127 | |
| 58 | IL-10RA | 0,200157 | 1,839836 | 1,87269 | -0,03285 | |
| 59 | IL4 | 0,200652 | 1,839836 | 1,774127 | 0,065708 | |
| 60 | AXIN1 | 0,2105 | 1,790554 | 4,13963 | -2,34908 | |
| 61 | ST1A1 | 0,2105 | 1,790554 | 4,13963 | -2,34908 | |
| 62 | IL6 | 0,21094 | 2,234086 | 1,839836 | 0,394251 | |
| 63 | uPA | 0,213468 | 1,839836 | 1,839836 | 0 | |
| 64 | Beta-NGF | 0,213478 | 1,839836 | 1,708419 | 0,131417 | |
| 65 | TRAIL | 0,216627 | 1,839836 | 3,646817 | -1,80698 | |
| 66 | CD40 | 0,226413 | 1,806982 | 4,76386 | -2,95688 | |
| 67 | LIF-R | 0,231209 | 1,839836 | 4,13963 | -2,29979 | |
| 68 | CCL28 | 0,236245 | 1,839836 | 1,774127 | 0,065708 | |
| 69 | IL7 | 0,237578 | 3,63039 | 1,839836 | 1,790554 | |
| 70 | CSF-1 | 0,241829 | 2,053388 | 1,839836 | 0,213552 | |
| 71 | TRANCE | 0,242016 | 4,13963 | 1,790554 | 2,349076 | |
| 72 | IL-17A | 0,244462 | 1,839836 | 4,13963 | -2,29979 | |
| 73 | IFN-gamma | 0,257088 | 1,839836 | 1,691992 | 0,147844 | |
| 74 | IL-22 RA1 | 0,263864 | 1,839836 | 1,806982 | 0,032854 | |
| 75 | ADA | 0,264653 | 3,63039 | 1,839836 | 1,790554 | |
| 76 | IL8 | 0,269508 | 1,659138 | 1,87269 | -0,21355 | |
| 77 | CCL11 | 0,269904 | 1,839836 | 4,13963 | -2,29979 | |
| 78 | TNFRSF9 | 0,27801 | 1,839836 | 1,823409 | 0,016427 | |
| 79 | CX3CL1 | 0,286338 | 2,234086 | 1,7577 | 0,476386 | |
| 80 | HGF | 0,291092 | 1,823409 | 5,38809 | -3,56468 | |
| 81 | CCL23 | 0,291092 | 1,823409 | 5,38809 | -3,56468 | |
| 82 | FGF-21 | 0,301031 | 1,839836 | 1,790554 | 0,049281 | |
| 83 | IL10 | 0,302058 | 2,004107 | 1,839836 | 0,164271 | |
| 84 | MMP-10 | 0,302448 | 1,806982 | 2,989733 | -1,18275 | |
| 85 | MCP-3 | 0,328359 | 1,544148 | 1,856263 | -0,31211 | |
| 86 | STAMBP | 0,328921 | 1,806982 | 3,186858 | -1,37988 | |
| 87 | SIRT2 | 0,328921 | 1,806982 | 3,186858 | -1,37988 | |
| 88 | CXCL6 | 0,349374 | 1,839836 | 1,839836 | 0 | |
| 89 | GDNF | 0,372651 | 1,839836 | 2,234086 | -0,39425 | |
| 90 | TNF | 0,427402 | 1,839836 | 1,839836 | 0 | |
| 91 | TGF-alpha | 0,45453 | 1,839836 | 1,856263 | -0,01643 | |
| 92 | IL33 | 0,675927 | 1,741273 | 1,87269 | -0,13142 | |

|  |  |  |  |  |  |
| --- | --- | --- | --- | --- | --- |
| **Supplementary Table 4**. Association of baseline circulating marker levels  with overall survival. | | | | | |
|  | Symbol | pval | Median OS High Value | Median OS Low value | Delta |
| 1 | PD-L1 | 0,018505 | 8,492813 | #N/A | #N/A |
| 2 | MCP-4 | 0,021631 | #N/A | 10,1848 | #N/A |
| 3 | IL-18R1 | 0,033182 | #N/A | 8,50924 | #N/A |
| 4 | MCP-2 | 0,077455 | #N/A | 10,61191 | #N/A |
| 5 | LAP TGF-beta-1 | 0,099017 | 8,50924 | #N/A | #N/A |
| 6 | LIF | 0,099356 | #N/A | 10,61191 | #N/A |
| 7 | CDCP1 | 0,099778 | 10,61191 | #N/A | #N/A |
| 8 | EN-RAGE | 0,103643 | 10,1848 | #N/A | #N/A |
| 9 | NRTN | 0,113683 | #N/A | 10,1848 | #N/A |
| 10 | CD5 | 0,132007 | #N/A | 10,61191 | #N/A |
| 11 | OPG | 0,140031 | #N/A | 10,61191 | #N/A |
| 12 | MMP-1 | 0,163005 | 7,227926 | #N/A | #N/A |
| 13 | CD6 | 0,17034 | 10,87474 | #N/A | #N/A |
| 14 | IL-17C | 0,178703 | #N/A | 3,843943 | #N/A |
| 15 | FGF-5 | 0,185592 | #N/A | 10,61191 | #N/A |
| 16 | uPA | 0,194372 | 10,61191 | #N/A | #N/A |
| 17 | NT-3 | 0,223292 | #N/A | 3,843943 | #N/A |
| 18 | CASP-8 | 0,229448 | 9,544148 | #N/A | #N/A |
| 19 | TNFB | 0,233604 | #N/A | 10,61191 | #N/A |
| 20 | IL8 | 0,237844 | 9,560575 | #N/A | #N/A |
| 21 | IL7 | 0,240668 | #N/A | 11,13758 | #N/A |
| 22 | OSM | 0,240668 | #N/A | 11,13758 | #N/A |
| 23 | FGF-19 | 0,26069 | 10,61191 | #N/A | #N/A |
| 24 | Beta-NGF | 0,288991 | #N/A | 10,61191 | #N/A |
| 25 | FGF-23 | 0,295085 | 10,61191 | #N/A | #N/A |
| 26 | CST5 | 0,32671 | #N/A | 11,13758 | #N/A |
| 27 | IL2 | 0,346249 | #N/A | 11,13758 | #N/A |
| 28 | CD40 | 0,346249 | 10,61191 | #N/A | #N/A |
| 29 | MCP-3 | 0,360175 | 10,61191 | #N/A | #N/A |
| 30 | CCL4 | 0,364503 | #N/A | 11,13758 | #N/A |
| 31 | ADA | 0,36869 | #N/A | 11,13758 | #N/A |
| 32 | IFN-gamma | 0,373088 | #N/A | 10,87474 | #N/A |
| 33 | TRAIL | 0,390687 | 11,13758 | #N/A | #N/A |
| 34 | TNFRSF9 | 0,392547 | 11,13758 | #N/A | #N/A |
| 35 | IL10 | 0,401133 | #N/A | 11,13758 | #N/A |
| 36 | TWEAK | 0,401745 | #N/A | 11,13758 | #N/A |
| 37 | HGF | 0,412299 | 10,61191 | #N/A | #N/A |
| 38 | CCL23 | 0,412299 | 10,61191 | #N/A | #N/A |
| 39 | CXCL9 | 0,412299 | 10,61191 | #N/A | #N/A |
| 40 | IL-20 | 0,414516 | #N/A | 8,50924 | #N/A |
| 41 | IL6 | 0,421151 | 10,1848 | #N/A | #N/A |
| 42 | SLAMF1 | 0,425195 | 10,87474 | #N/A | #N/A |
| 43 | Flt3L | 0,439219 | #N/A | 10,61191 | #N/A |
| 44 | IL-10RA | 0,451865 | 10,87474 | #N/A | #N/A |
| 45 | CXCL1 | 0,473757 | 10,61191 | #N/A | #N/A |
| 46 | TGF-alpha | 0,503746 | 10,61191 | #N/A | #N/A |
| 47 | CSF-1 | 0,525179 | 10,1848 | #N/A | #N/A |
| 48 | CCL11 | 0,565213 | #N/A | 11,13758 | #N/A |
| 49 | CXCL6 | 0,57189 | #N/A | 11,13758 | #N/A |
| 50 | GDNF | 0,590466 | 10,61191 | #N/A | #N/A |
| 51 | IL5 | 0,612813 | 10,61191 | #N/A | #N/A |
| 52 | CX3CL1 | 0,613819 | #N/A | 11,13758 | #N/A |
| 53 | CCL19 | 0,613819 | #N/A | 11,13758 | #N/A |
| 54 | MCP-1 | 0,618275 | #N/A | 11,13758 | #N/A |
| 55 | AXIN1 | 0,640054 | 10,61191 | 11,13758 | -0,52567 |
| 56 | ST1A1 | 0,640054 | 10,61191 | 11,13758 | -0,52567 |
| 57 | IL-22 RA1 | 0,646741 | #N/A | 11,13758 | #N/A |
| 58 | IL-17A | 0,661834 | #N/A | 11,13758 | #N/A |
| 59 | TSLP | 0,678533 | 11,13758 | #N/A | #N/A |
| 60 | IL-10RB | 0,678745 | 11,13758 | #N/A | #N/A |
| 61 | VEGFA | 0,699043 | 10,61191 | #N/A | #N/A |
| 62 | CD8A | 0,701266 | 11,13758 | 10,1848 | 0,952772 |
| 63 | CD244 | 0,701266 | #N/A | 11,13758 | #N/A |
| 64 | DNER | 0,704785 | 11,13758 | 3,843943 | 7,293634 |
| 65 | CCL28 | 0,723713 | #N/A | 11,13758 | #N/A |
| 66 | IL-12B | 0,755629 | #N/A | 11,13758 | #N/A |
| 67 | IL-15RA | 0,778731 | 11,13758 | 10,1848 | 0,952772 |
| 68 | IL13 | 0,797814 | #N/A | 11,13758 | #N/A |
| 69 | TNFSF14 | 0,805426 | 10,61191 | #N/A | #N/A |
| 70 | CXCL5 | 0,809352 | #N/A | 11,13758 | #N/A |
| 71 | IL18 | 0,810038 | 11,13758 | 10,61191 | 0,525667 |
| 72 | SCF | 0,812637 | 11,13758 | #N/A | #N/A |
| 73 | IL-2RB | 0,81651 | 11,13758 | #N/A | #N/A |
| 74 | CXCL11 | 0,822382 | 8,50924 | #N/A | #N/A |
| 75 | IL33 | 0,83998 | 10,61191 | #N/A | #N/A |
| 76 | IL-24 | 0,847379 | 10,61191 | 11,13758 | -0,52567 |
| 77 | IL-20RA | 0,852223 | 11,13758 | #N/A | #N/A |
| 78 | CCL25 | 0,863819 | 8,50924 | 11,13758 | -2,62834 |
| 79 | TRANCE | 0,869679 | 11,13758 | #N/A | #N/A |
| 80 | TNF | 0,870879 | 10,1848 | #N/A | #N/A |
| 81 | CCL3 | 0,875816 | 10,61191 | 11,13758 | -0,52567 |
| 82 | 4E-BP1 | 0,909635 | 8,50924 | 11,13758 | -2,62834 |
| 83 | LIF-R | 0,911397 | #N/A | 11,13758 | #N/A |
| 84 | CCL20 | 0,926826 | #N/A | 11,13758 | #N/A |
| 85 | CXCL10 | 0,926899 | #N/A | 11,13758 | #N/A |
| 86 | IL-1 alpha | 0,926899 | 11,13758 | #N/A | #N/A |
| 87 | ARTN | 0,941089 | 11,13758 | #N/A | #N/A |
| 88 | IL4 | 0,966009 | 11,13758 | #N/A | #N/A |
| 89 | FGF-21 | 0,96851 | 11,13758 | 10,61191 | 0,525667 |
| 90 | STAMBP | 0,970732 | #N/A | 11,13758 | #N/A |
| 91 | SIRT2 | 0,970732 | #N/A | 11,13758 | #N/A |
| 92 | MMP-10 | 0,988466 | #N/A | 11,13758 | #N/A |

**SUPPLEMENTARY FIGURES**

**
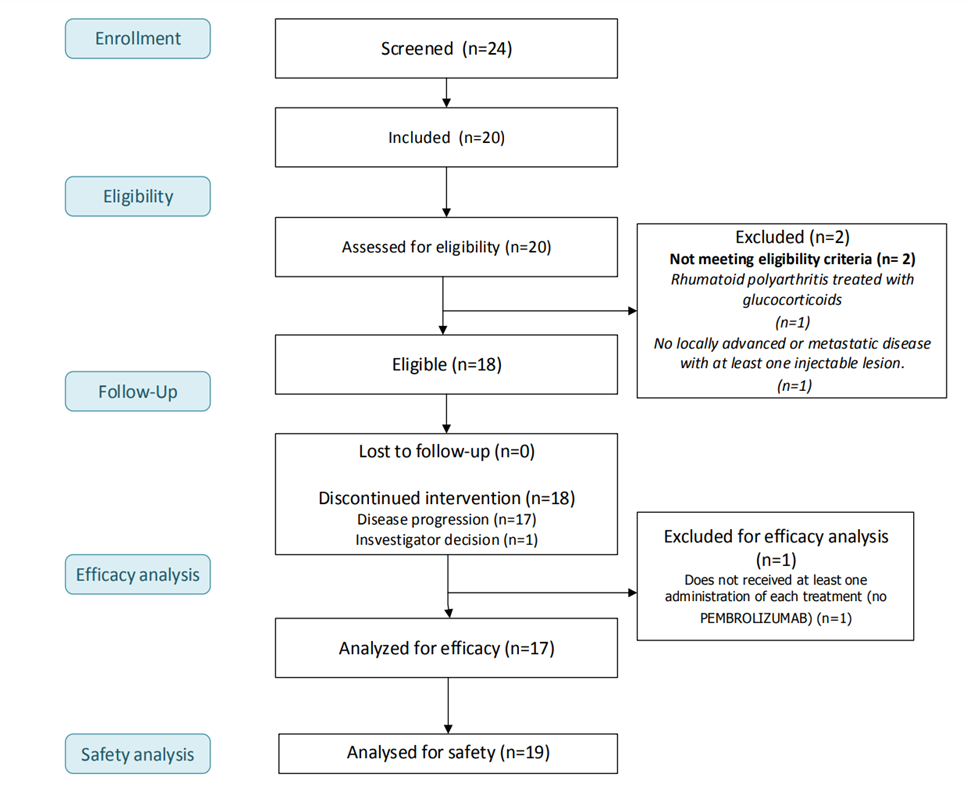
**

**Supplementary Figure 1.** Flow-chart of the study.

**
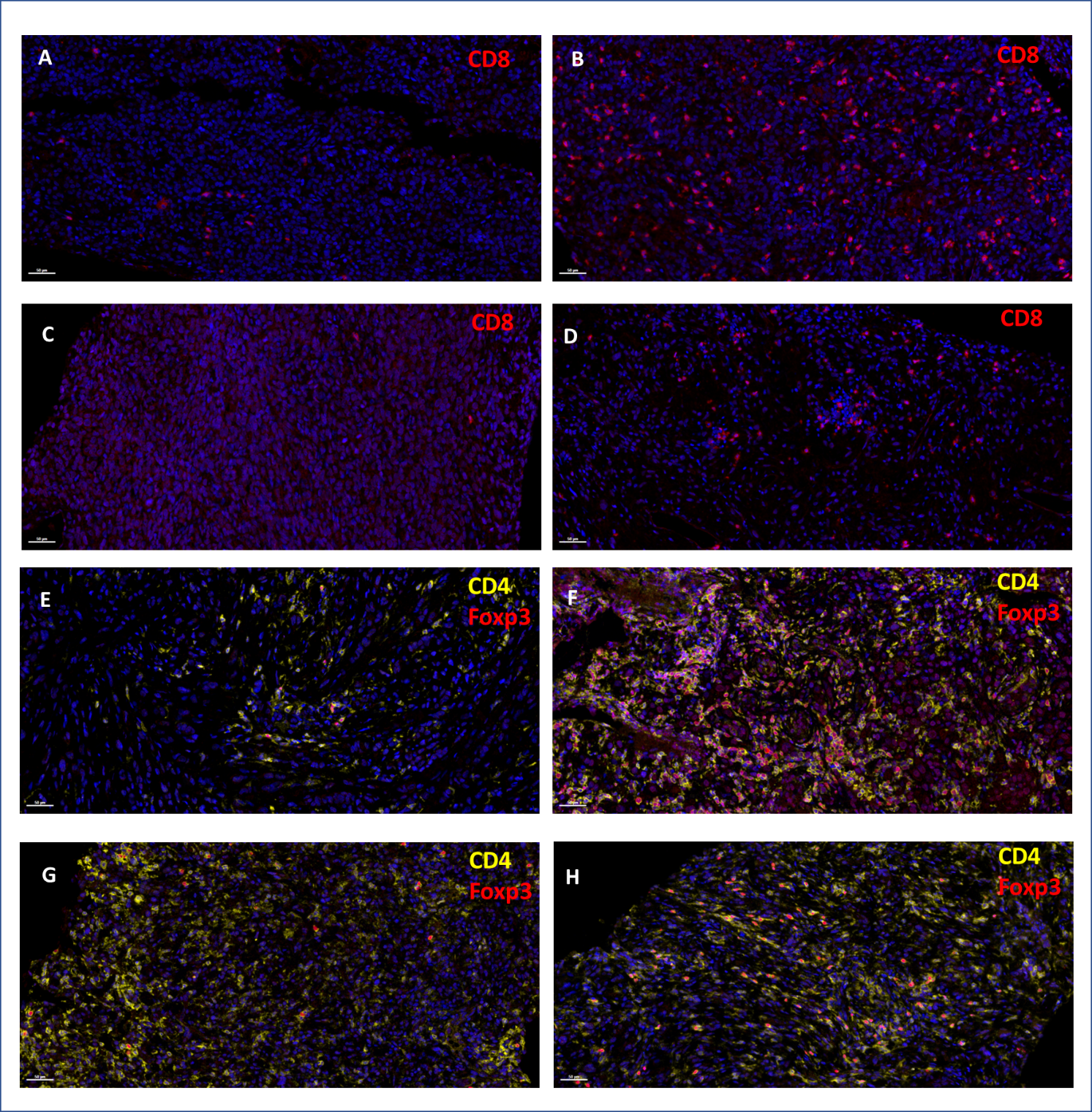
**

**Supplementary Figure 2. Characterization of the impact of intra-tumoral injection of G100 on the microenvironment of TLS-negative soft-tissue sarcomas**. Cases (n = 2) stained with the multiplex panel CD8/GzmA/CD4/FOXP3/CD56. Illustration of increase in CD8+ T cells (B,D) and CD4+/FoxP3+ T cells (F,H) infiltration at cycle 2 (B,D, F,H) versus baseline (A,C,E,G)


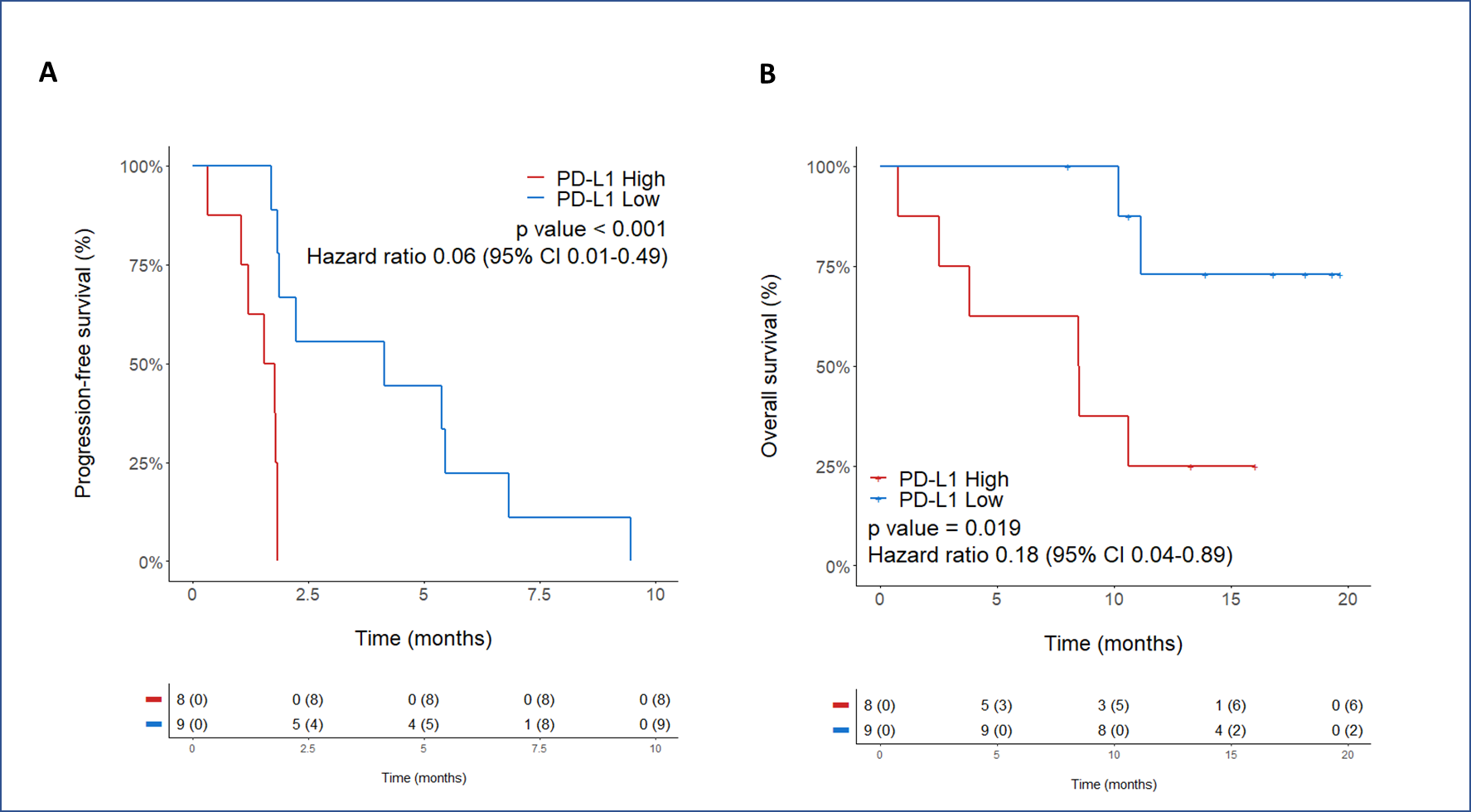


**Supplementary Figure 3. Kaplan-Meir curves of progression-free survival (A) and overall survival (B) according to baseline soluble PD-L1 levels (blue curve: low, red curve: high, n=17)**
